# The complete mitochondrial genome of the Picasso clownfish: genomic comparisons and phylogenetic inference among Amphiprioninae

**DOI:** 10.1080/23802359.2020.1797554

**Published:** 2020-07-27

**Authors:** Li-bin He, Shui-qing Wu, Hui-yu Luo, Le-yun Zheng

**Affiliations:** a Center for prevention and control of marine biological diseases, Fisheries Research Institute of Fujian, Xiamen, China; b Marine Biological Genetics and Breeding Research Center, Fisheries Research Institute of Fujian, Xiamen, China

**Keywords:** Picasso clownfish, next-generation sequencing, mitochondrial genome, phylogenetic relationships, comparative analysis

## Abstract

Picasso clownfish belong to the subfamily Amphiprioninae and are considered a variant of the genus *Amphiprion*. In this study, we first sequenced the complete mitochondrial genome of the Picasso clownfish by Illumina next-generation sequencing technology. The length of the whole mitogenome is 16,727 bp long, with a gene arrangement and composition similar to those of two other *Amphiprion* species (*Amphiprion ocellaris* and *Amphiprion percula*). The topological structure of the phylogenetic tree shows that the Picasso clownfish is more closelyrelated to *A. percula* than it is to *A. ocellaris*, suggesting that the Picasso clownfish may be a variant of *A. percula*.

The subfamily Amphiprioninae (family Pomacentridae), which includes approximately 30 species (Allen 1991; Allen et al. 2008, 2010), is well known as the anemonefishes because these species form mutualistic symbiotic relationships with tropical sea anemones (Elliott et al. 1999). The Picasso clownfish,an anemonefish variety named for its irregular body stripes,still lacks a Latin name. In the present study, wedetermined the complete mitochondrial genome of the Picasso clownfish using next-generation Illumina sequencing technology and analyzed the phylogenetic relationships within the Amphiprioninae.

The Picasso clownfish were collected from Fangcun aquariums in Guangzhou (23°6′15″N, 113°26′29″E), Guangdong Province, China. The specimen was deposited at the Culture Collection of Fish at Fisheries Research Institute of Fujian,Sample Number:BJS17928. A 30–40 mg fin clip was preserved in 95% ethanol immediately and stored at −20 °C until DNA extraction. Total genomic DNA was extracted using the DNeasy Tissue Kit (Qiagen, Germany) according to the manufacturer's protocol. Adapter-modified DNA fragments were PCR-amplified using PE PCR primers. Libraries were sequenced using an Illumina HiSeq4000 at BGI-Shanghai, China, with 6 Gb of 2 × 150-bp paired-ends,which was constructed with two indexes using the Illumina TruSeq@ DNA PCR-Free HT Kit. Finally, the complete mitochondrial genome sequence was submitted to GenBank with accession number SRR6363367.

The complete mitogenome (16,727bp) of the Picasso clownfish, which was longer than those of the other two *Amphiprion* species(*Amphiprion ocellaris* and *Amphiprion percula*), contained 13 PCGs, 22 tRNA genes, two rRNA genes and two non-coding regions. From the complete mitochondrial genome, the gene arrangement and translation direction were basically identical to those in *A. percula* and *A. ocellar* is (Figure 1). The mitochondrial genome of the Picasso clownfish consisted of A = 29.2%, T = 25.8%, G = 16.0% and C = 29.00% (AT-skew = 0.06 and GC-skew = −0.29).

**Figure 1.**
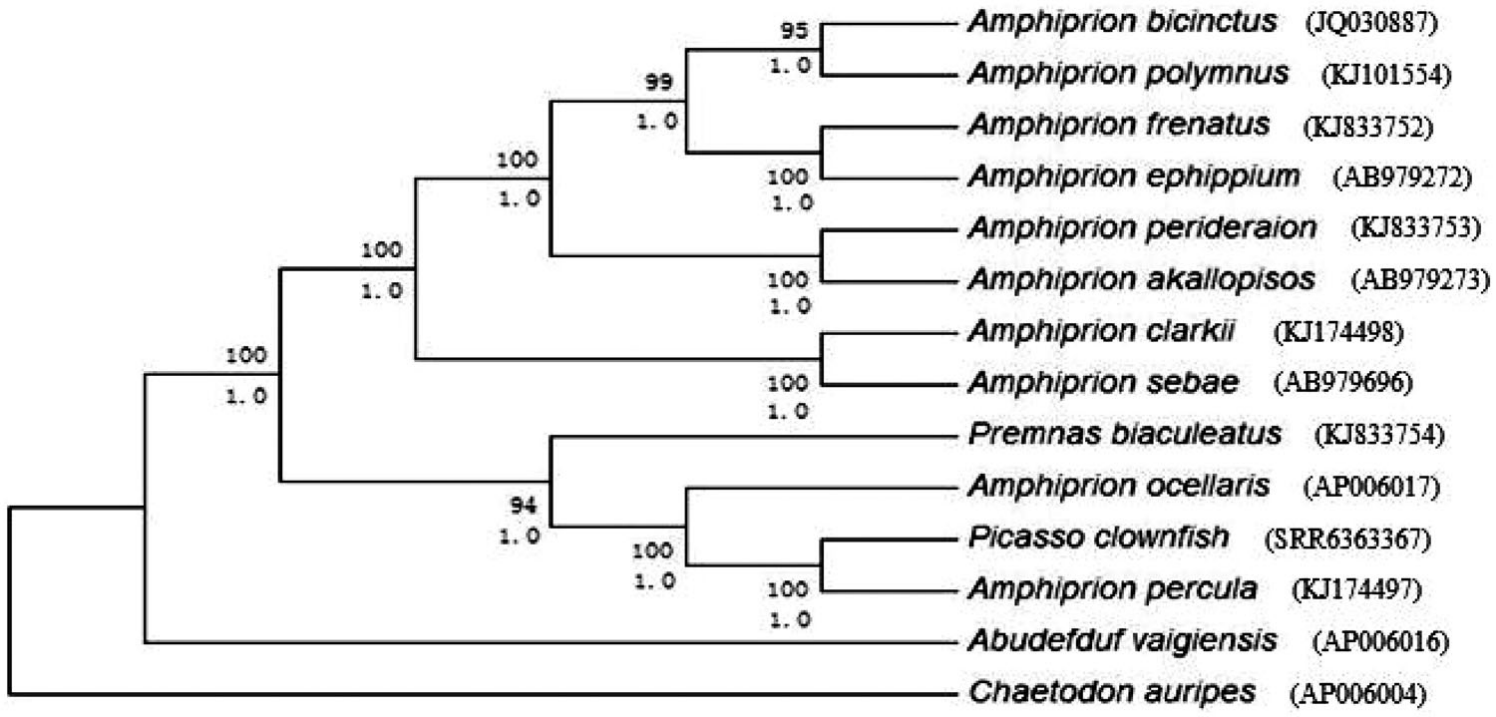
Phylogenetic trees derived from neighbour-joining (NJ) and Bayesian inference (BI) analyses based on nucleotide sequences of 13 mitochondrial protein-coding genes and 2 ribosomal RNA genes. Tree topologies produced by BI and NJ analyses were equivalent. Bayesian posterior probability (bottom) and bootstrap support values for NJ analyses (top) are shown in order on the nodes. *Chaetodon auripes* was selected as an outgroup species.

The BI and NJ trees based on 2 rRNAs and 13 PCGs showed similar topologies and branch support within Amphiprioninae, andthe support values were robust,with 90% bootstrap values (Figure 1). The anemonefishes in Amphiprioninae are separated into two clades. Clade I includes *Amphiprion bicinctus, Amphiprion clarkii, Amphiprion frenatus, Amphiprion polymnus, Amphiprion perideraion, Amphiprion sebae, Amphiprion ephippium* and *Amphiprion akallopisos* and corresponds well with a previous study based on morphological traits (Allen 1972, 1975). The support values for these clades were very high and stable for both analyses. In Clade II, we found that *Premnas biaculeatus, Aocellaris, A. percula* and the Picasso clownfish clustered in one branch of the phylogenetic tree with high support values and that the Picasso clownfish was more closely related to *A. percula* than it was to *A.ocellaris*. Our study corroborates the relationship between *A. percula* and the Picasso clownfish, namely, we suggesting that the Picasso clownfish may be a variant of *A. percula*.

## Data Availability

The data that support the findings of this study are openly available in GenBank database at https://submit.ncbi.nlm.nih.gov/subs/sra/SUB3297187/overview, reference number [SRR6363367].

## References

[CIT0001] Allen GR. 1972. The Anemonefishes: their classification and biology. Neptune City, NJ, USA: Tropical Fish Hobbyist Publications; p. 1–288

[CIT0002] Allen GR. 1975. The Anemonefishes: their classification and biology. 2nd ed. Neptune City, New Jersey: Tropical Fish Hobbyist Publications; p. 1–352.

[CIT0003] Allen GR. 1991. Damselfishes of the world. Melle: Mergus Publishers.

[CIT0004] Allen GR, Drew J, Kaufman L. 2008. *Amphiprion barberi*, a new species of anemonefish (Pomacentridae) from Fiji, Tonga, and Samoa. Aqua Int J Ichthyol. 14:105–114.

[CIT0005] Allen GR, Drew J, Fenner D. 2010. *Amphiprion pacificus*, a new species of anemonefish (Pomacentridae) from Fiji, Tonga, Samoa, and Wallis Island. Aqua Int J Ichthyol. 16:129–138.

[CIT0006] Elliott JK, Lougheed SC, Bateman B, McPhee LK, Boag PT. 1999. Molecular phylogenetic evidence for the evolution of specialization in anemonefishes. Proc Biol Sci. 266(1420):677–685. 10331288 10.1098/rspb.1999.0689 PMC1689819

